# The vaginal microbiome during pregnancy and the postpartum period in a European population

**DOI:** 10.1038/srep08988

**Published:** 2015-03-11

**Authors:** David A. MacIntyre, Manju Chandiramani, Yun S. Lee, Lindsay Kindinger, Ann Smith, Nicos Angelopoulos, Benjamin Lehne, Shankari Arulkumaran, Richard Brown, Tiong Ghee Teoh, Elaine Holmes, Jeremy K. Nicoholson, Julian R. Marchesi, Phillip R. Bennett

**Affiliations:** 1 Imperial College Parturition Research Group, Division of the Institute of Reproduction and Developmental Biology, Imperial College London, UK; 2 School of Biosciences, Cardiff University, UK; 3 Division of Cancer, Department of Surgery and Cancer, Imperial College London, UK; 4 Department of Epidemiology & Biostatistics, Medicine, Imperial College London, UK; 5 St Mary's Hospital, Imperial College Healthcare NHS Trust, London, UK; 6 Section of Biomolecular Medicine, Division of Computational Systems Medicine, Department of Surgery and Cancer, Imperial College London, UK; 7 MRC NIHR National Phenome Centre, Division of Computational Systems Medicine, Department of Surgery and Cancer, Imperial College London, UK

## Abstract

The composition and structure of the pregnancy vaginal microbiome may influence susceptibility to adverse pregnancy outcomes. Studies on the pregnant vaginal microbiome have largely been limited to Northern American populations. Using MiSeq sequencing of 16S rRNA gene amplicons, we characterised the vaginal microbiota of a mixed British cohort of women (n = 42) who experienced uncomplicated term delivery and who were sampled longitudinally throughout pregnancy (8–12, 20–22, 28–30 and 34–36 weeks gestation) and 6 weeks postpartum. We show that vaginal microbiome composition dramatically changes postpartum to become less *Lactobacillus* spp. dominant with increased alpha-diversity irrespective of the community structure during pregnancy and independent of ethnicity. While the pregnancy vaginal microbiome was characteristically dominated by *Lactobacillus* spp. and low alpha-diversity, unlike Northern American populations, a significant number of pregnant women this British population had a *L. jensenii*-dominated microbiome characterised by low alpha-diversity. *L. jensenii* was predominantly observed in women of Asian and Caucasian ethnicity whereas *L. gasseri* was absent in samples from Black women. This study reveals new insights into biogeographical and ethnic effects upon the pregnancy and postpartum vaginal microbiome and has important implications for future studies exploring relationships between the vaginal microbiome, host health and pregnancy outcomes.

The vaginal microbiome in pregnancy plays an important role in both maternal and neonatal health outcomes. Pregnancy is accompanied by a shift in the bacterial community structure of the vagina to a composition that is typically dominated by one or two species of *Lactobacillus*^1^,^2^,^3^,^4^,^5^. These bacteria are believed to inhibit pathogen growth through secretion of antibacterial bacteriocins as well as the production of metabolites such as lactic acid that help to maintain a low, hostile pH^6^. Dysbiosis of the vaginal microbiome is associated with complications of pregnancy, in particular an increased risk of preterm birth^7^,^8^,^9^,^10^,^11^. The maternal vaginal microbiome may also be an important source of pioneer bacteria for the neonatal gut microbiome^12^,^13^, which have a profound effect on host system metabolism and immunity^14^,^15^,^16^.

In asymptomatic non-pregnant North American women of reproductive age, five vaginal bacterial community state types (CSTs) have been defined^17^. Four of these are dominated by *Lactobacillus* species including *L. crispatus* (CST I), *L. gasseri* (CST II), *L. iners* (CST III), and *L. jensenii* (CST V). CST IV is typically characterised by low levels of *Lactobacillus* spp. and increased diversity of anaerobic bacteria including *Prevotella, Dialister, Atopobium vaginae, Gardnerella vaginalis, Megasphaera, Peptoniphilus, Sneathia, Finegoldia*, and *Mobiluncus*. The latter species are often associated with bacterial vaginosis, a clinical syndrome of vaginal discharge and odour characterised by polymicrobial overgrowth, which is linked to an increased risk of preterm birth^8^,^9^ and histological chorioamnionitis^11^,^18^,^19^. Interestingly, in American populations, vaginal bacterial communities dominated by *Lactobacillus* spp. (CST I, II, III and V) are most frequently observed in Asian and White women whereas a diverse microbiome (CST IV) is more frequently observed in Black and Hispanic populations suggesting that the composition of the vaginal microbiome may be, in part, shaped by genetic differences between hosts but cultural and behavioural factors cannot be excluded to explain these associations^17^,^20^. These findings have been recently confirmed and extended by Fettweis and colleagues who identified clear ethnic related differences in the vaginal microbiome of a large population of healthy Black and White Northern American women^21^.

The vaginal microbiome during pregnancy has recently been examined in cross–sectional^1^ and longitudinal^3^,^22^ patient cohorts derived from North American based populations. These studies confirm that the vaginal microbiome becomes less diverse and more stable during pregnancy and is largely dominated by *Lactobacillus* spp. Whilst they have pointed towards a general reduction of species diversity during pregnancy, the distribution of vaginal bacteria community state types appears to vary by population. A recent study by Hyman et al (2014) indicates that ethnicity may be an important determinant of specific aspects of the vaginal microbial composition during pregnancy^23^. Data from the Vaginal Microbiome Consortium supports this with microbial taxa associated with preterm birth, particularly *Prevotella* spp. and *Sneathia* spp., shown to be more prevalent in the microbiomes of Black Northern American women compared to White Northern American women^21^.

While much attention has been focused upon the vaginal microbiome during pregnancy, there is a distinct lack of information regarding the vaginal microbiome in the post partum period. This oversight is surprising considering the association of anaerobic vaginal microbiota and post-delivery pathologies such as postpartum endometritis^24^,^25^ and sepsis^26^. Culture based methods have shown early postpartum endometritis is typically characterised by the presence of multiple microbiota often associated with bacterial vaginosis including *Gardnerella vaginalis*, *Peptococcus* spp., *Bacteroides* spp., *Staphylococcus epidermidis*, *Streptococcus agalactiae* and *Ureaplasma urealyticum*^27^.

In this study we aimed to examine the composition of the vaginal microbiome throughout pregnancy and in the postpartum period. We hypothesised that there exists differences in the vaginal pregnancy microbiome of the United Kingdom and North America populations. We show that the vaginal microbiome dramatically changes in the post partum period irrespective of ethnicity to become less *Lactobacillus* spp. dominant with greater alpha-diversity irrespective of the community structure for that individual. Consistent with previous studies, we show that our British population demonstrates a pregnancy vaginal microbiome characterised by *Lactobacillus* spp. dominance and low alpha-diversity. However, unlike women in North American populations, significant numbers of White and Asian British women have an *L. gasseri* (CST V) dominated microbiome that is also associated with low alpha-diversity.

## Methods

### Volunteers and samples

This study was undertaken following NHS Health Research Authority National Research Ethics Service (NRES) Committee Approval (REC 11/EM/0097). All experiments were performed in accordance with the approved guidelines. Informed consent was obtained from all subjects prior to sampling. Women (n = 42) with an uncomplicated singleton pregnancy, who had no medical problems or adverse outcomes during any previous pregnancy, were recruited to the study at booking of their antenatal care. Samples were collected under direct visualisation during a speculum examination from the posterior fornix by using a BBL™ CultureSwab™ MaxV Liquid Amies swab (Becton, Dickinson and Company, Oxford, UK), at gestational ages 8–12, 19–25, 27–30 and 32–36 weeks and then six weeks post delivery. Vaginal swab samples were immediately frozen and stored at −80°C until extraction. Detailed medical and gynaecological history was taken by the research team, in particular to include information including time since last sexual intercourse and douching practices. Exclusion criteria included women who had sexual activity within 72 h of sampling, reported vaginal bleeding in the preceding week, used antibiotics in the preceding 2 weeks, multiple pregnancies, were HIV positive or under the age of 18 years. Women were eliminated from the study if they underwent either spontaneous or indicated preterm delivery prior to 37 weeks, or if they developed any inter-current infection requiring antibiotic therapy. Ethnicity was self-reported as White (European ancestry), Black (African or African-Caribbean ancestry) or Asian (Pakistani, Indian, Bangladeshi or Sri Lankan ancestry).

### DNA extraction

Swabs were thawed on ice and re-suspended in transport buffer (Amies Liquid Medium) by vortexing. Cells were transferred to a sterile DNase/RNase free 2 ml tube where an enzymatic lysis step was carried out for 1 h at 37°C as previously described^17^. Samples underwent additional mechanical disruption by using a Mikro-Dismembrator (Sartorius UK Ltd, Surrey, United Kingdom) with acid washed glass beads for 1 min at 1000 x *g*. The resulting lysate was further processed and purified using QIAamp DNA Mini kit (Qiagen, Manchester, UK) and the DNA was eluted in 100 μl AE buffer. The integrity of the extracted bacterial DNA was confirmed by PCR amplification using the universal primers: 27F-5′-AGAGTTTGATCCTGGCTCAG-3′ and 338R-5′-GCTGCCTCCCGTAGGAGT-3′^28^.

### MiSeq sequencing

The V1-V2 hypervariable regions of 16S rRNA genes were amplified for sequencing using a forward and reverse fusion primer. The forward primer was constructed with the Illumina i5 adapter (5′-3′) (AATGATACGGCGACCACCGAGATCTACAC), an 8–10 bp barcode, a primer pad (Forward: TATGGTAATT), and the 28F-GAGTTTGATCNTGGCTCAG primer^29^. The reverse fusion primer was constructed with (5′-3′) the Illumina i7 adapter (CAAGCAGAAGACGGCATACGAGAT), an 8–10 bp barcode, a primer pad (Reverse: AGTCAGTCAG), and the reverse primer (388R-TGCTGCCTCCCGTAGGAGT)^30^. Primer pads were designed to ensure the primer pad/primer combination had a melting temperature of 63°C–66°C according to methods developed by the lab of Patrick Schloss (http://www.mothur.org/w/images/0/0c/Wet-lab_MiSeq_SOP.pdf). Amplifications were performed in 25 μl reactions with Qiagen HotStar Taq master mix (Qiagen Inc, Valencia, California), 1 μl of each 5 uM primer, and 1 μl of template. Reactions were performed on ABI Veriti thermocyclers (Applied Biosytems, Carlsbad, California) under the following thermal profile: 95°C for 5 min, then 35 cycles of 94°C for 30 sec, 54°C for 40 sec, 72°C for 1 min, followed by one cycle of 72°C for 10 min and 4°C hold. Amplification products were visualized with eGels (Life Technologies, Grand Island, New York). Products were then pooled equimolar and each pool was size selected in two rounds using Agencourt AMPure XP (BeckmanCoulter, Indianapolis, Indiana) in a 0.7 ratio for both rounds. Size selected pools were then quantified using the Quibit 2.0 Fluorometer (Life Technologies) and loaded on an Illumina MiSeq (Illumina, Inc. San Diego, California) 2 × 300 flow cell at 10 pM. All sequencing was performed at Research and Testing Laboratory (Lubbock, TX, USA).

### Sequence analysis

The 16S rRNA gene sequences generated were analysed using the bioinformatic software package Mothur^31^ using the MiSeq SOP Pipeline to analyse a multiplexed set of samples on a single run. The paired reads were assembled using make.contigs that extract the sequences and quality score data from the fastq files, and creates the reverse complement of the reverse read and finally assembles the paired end reads into a contig. Screen.seqs was used to remove low quality reads using the following filtering parameters, maxn = 0, maxambig = 0, maxhomop = 5, minlength = 307 and maxlength = 339. The kmer searching method was used to align our sequences by using a Silva bacterial database (www.arb-silva.de/), with the flip parameter set to true, which allows the reverse complement of the sequence to be aligned for better results. The screen.seqs command was implemented again to keep within our defined criteria, using the following parameters: start = 1044, end = 6333 and maxhomop = 5. The filter.seqs was used to remove empty columns from our alignment, which gives our length of filtered alignment to 698. To classify (classify.seqs) our sequences we used a RDP database/reference sequence files and used the Wang method^32^. Finally to normalise (sub.sample) our data we used the smallest set of reads (1916). 16S rRNA gene sequence reads were quality checked and normalised to the lowest number of reads in Mothur. Singleton OTUs and those not found more than 10 times in any sample were collated into OTU_singletons and OTU_rare phylotypes respectively, to maintain normalisation and to minimise artefacts^33^. Using the Vegan package within the R statistical package^34^, analysis was performed on the datasets contained within the files generated by Mothur (all OTUs were defined using a cut off value of 97%). The Unifrac weighted distance matrix was analysed in R using non-metric multidimensional scaling ordination and the shared OTU file was used to determine the number of times that an OTU is observed in multiple samples, and was used for multivariate analysis in R. OTU taxonomies (from Phylum to Genus) were determined using the RDP MultiClassifier script to generate the RDP taxonomy. In order to obtain species level taxonomies of the OTUs, USEARCH was used with 16S rRNA gene sequences from the cultured representatives from the RDP database^35^. Alpha and beta indices were calculated from these datasets with Mothur and R using the Vegan package. The Simpson index was used as a measurement of alpha-diversity as it takes into account both species richness and the evenness of abundance among the species present in a given sample^36^. We report the inverse value of the classical Simpson estimate as a measure of alpha diversity as it provides a more intuitive interpretation where a higher value indicates higher diversity.

### Statistical analyses

To determine statistical differences between the vaginal microbiome throughout gestation and post partum, the Statistical Analysis of Metagenomic Profiles (STAMP) software package was used^37^. Data were subjected to multivariate analysis in the form of unsupervised principal components analysis (PCA) to assess correlated variance in the dataset. Hierarchical clustering analysis was performed using centroid linkage with a clustering density threshold of 0.75. *P*-values were calculated using Welch's t-test^38^ with multiple testing corrections applied using the Benjamini–Hochberg false discovery rate^39^. A *P*-value < 0.05 and a *q*-value < 0.05 was considered significant. Non-metric multidimensional scaling (NMDS) was used to visualise the pairwise UniFrac distances among samples. Modelling of the vaginal community state types as a function of gestation age was performed using in-house scripts in Swi-Prolog^40^, which uses Real^41^ to access primitive graphics call in R.

To assess the statistical significance of microorganism abundances and CST during and after pregnancy, we used linear mixed model regression analysis. Analyses were performed in R using the R package, lme4 (R package version 1.1–7, http://CRAN.R-project.org/package=lme4). For each analysis false discovery rate adjustment (Benjamin & Hochberg) was applied to correct *P*-values. In total four analyses were carried out as follows. i) To test if bacterial species abundance changes significantly over the course of pregnancy we excluded postpartum measurements and performed a linear regression for each microorganism. Abundances were log-transformed and regressed against time and adjusted for ethnicity and patient ID, whereby patient ID is modelled as a random effect to account for the correlation between samples of the same individual. ii) To examine if microorganism abundance changes significantly after pregnancy, a binary time variable was created with time = 0 for measurements during pregnancy and time = 1 for postpartum measurements. Using this binary time variable we performed a linear regression for each microorganism. Abundances were log-transformed and regressed against time adjusted for ethnicity and patient ID, whereby patient ID is modelled as a random effect. iii) To test if CSTs change significantly over the course of pregnancy we excluded postpartum measurements and performed a linear regression for each CST. A CST indicator variable was created where CST = 1 for samples that could be assigned to the given CST and CST = 0 for all other samples. Time was regressed against CST adjusted for ethnicity and patient ID, whereby patient ID is modelled as a random effect. iv) To assess if CSTs change significantly after pregnancy, a binary time variable was created with time = 0 for measurements during pregnancy and time = 1 for postpartum measurements and linear regression performed for each CST. A CST indicator variable was generated whereby CST = 1 for samples that could be assigned to the given CST and CST = 0 for all other samples. Time was regressed against CST adjusted for ethnicity and patient ID, whereby patient ID is modelled as a random effect.

## Results

### The vaginal microbial community composition is significantly altered in the postpartum period

A total of 42 women were recruited into the study. Of these, 11 attended for sampling at all time points including sampling 6 weeks postpartum. During pregnancy all but one subject attended for at least three of the four time points, however only 15 attended for postnatal sampling. Subjects were self-identified into three ethnic groups; White (n = 23), Black (n = 5) or Asian (n = 13). A total number of 2113251 reads were obtained from 157 samples. The average number of reads per sample was 14088 and the mean and median read lengths were 331 and 332 bp respectively. The lowest read count was 1916 and therefore, OTUs were randomly sub-sampled to 1916 reads for further analysis to avoid sequencing bias. When singletons and rare OTUs were removed from the data set, a total of 47 taxa were identified in the vaginal microbiome of the study population (Supplementary Dataset Table 1).

**Figure 1:**
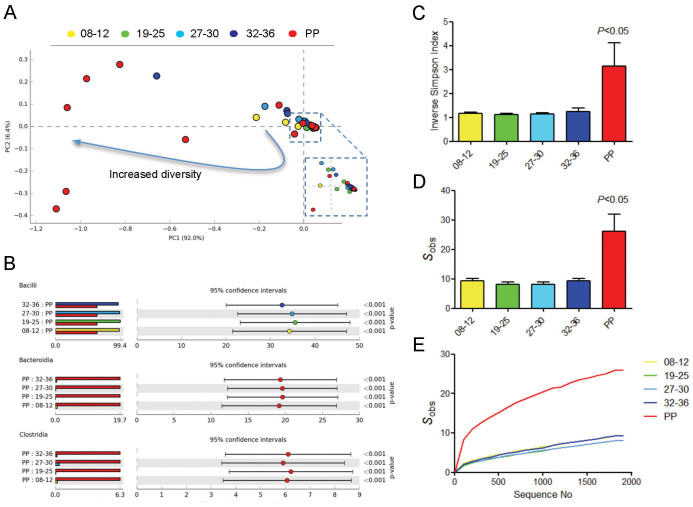
Bacterial class diversity is significantly increased postpartum. (A) Principal component analysis of the vaginal bacterial classes during gestation and 6 weeks postpartum shows that post pregnancy is associated with a marked shift in the microbiome in a high proportion of sampled women. The majority of relative bacterial class abundance variation in the data set was described by the first two principal components (PC; PC1 = 92%, PC2 = 6.4%). (B) Lowest diversity was observed in samples collected from women mid-gestation with a significant increase diversity seen between 32–36 weeks gestation. A significant increase in diversity was observed postpartum determined by ANOVA with Tukey-Kramer post hoc test using a Benjamini-Hochberg FDR correction. Data is presented at mean proportions of total sequence data (left side) and differences in mean proportions (right side) compared to postpartum samples. (C) The vaginal microbiome postpartum is characterised by a significant decrease in the Bacilli class of bacteria as well as proportional increased in Clostridia, Bacteroidia and Actinobacteria classes. (D) The postpartum period was also associated with increased richness as determined by the average number of species observed and (E) as a function of sequence depth as assessed using a rarefraction curve.

The vaginal community structure during pregnancy and post partum period was firstly assessed by PCA of relative class abundances (Figure 1A). While pregnancy was associated with a microbiome largely dominated by Bacilli, the postpartum period was characterised by marked shift in bacterial class structure. A similar pattern was observed at the level of genera (Supplementary Figure 1). The mean proportion of Bacilli was significantly reduced postpartum by approximately 35% when compared to any times points collected during pregnancy (*q* < 0.001) (Figure 1B). The postpartum period was also accompanied by increases in bacteria from the classes Clostridia (~6% increase, *P* < 0.001) and Bacteriodia (~20% increase, *P* < 0.001). Consistent with these observations, indices of alpha-diversity and richness were least in samples obtained during mid gestation with a significant increase in diversity (*P* < 0.05) detected in samples obtained 6 weeks postpartum (Figure 1C, D and E).

### Characteristics and dynamics of the bacterial species composition of vaginal community state types throughout pregnancy and postpartum

**Figure 2:**
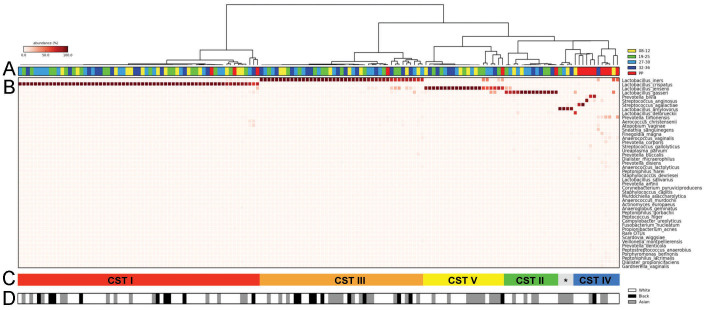
Bacterial species composition of vaginal community state types (CST) throughout pregnancy and postpartum. (A) Hierarchical clustering analysis using centroid linkage of microbial species data shows that vaginal microbiomes from a UK cohort can be clustered into 5 major groups consistent with vaginal CSTs previously identified in non-pregnant and pregnant North American populations. Around 75% of all postpartum samples were found to cluster into CST-IV. (B) Heatmap of relative abundances of bacterial species characterising the CSTs. (C&D) CSTs I, III and IV were represented by similar proportions of White, Asian and Black ethnicities however CST II and V were almost void of representation from black women. CST * indicates *Lactobacillus amylovorous* dominated microbiome.

Hierarchical clustering analysis of bacterial species from the pregnant and postpartum vaginal microbiome communities revealed 5 major groups that reflect vaginal bacterial community state types (CSTs) previously defined in non-pregnant, reproductive age North American populations^17^ (Figure 2). This analysis was performed using all samples obtained from women recruited to our study cohort. The most commonly observed CST was CST I (*L. crispatus*, 40%), followed by CST III (*L. iners*, 27%), CST V (*L. jensenii*, 13%) and CST II (*L. gasseri*, 9%), respectively. CST IV was characterised by reduced *Lactobacillus* spp. and increased proportion of bacterial species associated with bacterial vaginosis including *Prevotella* spp., *Clostridium* spp., *Atopobium* spp., and *Megasphaera* spp and was observed in 8% of all samples. However CST IV was rarely observed in pregnant samples (3/142, 2%). Four samples isolated from the same woman were dominated by *Lactobacillus amylovorous* (3%). Further phylogenetic analysis was undertaken (data not shown) to verify that the taxonomic position of this sequence and confirm that it does cluster with *L. amylovorous*.

**Figure 3:**
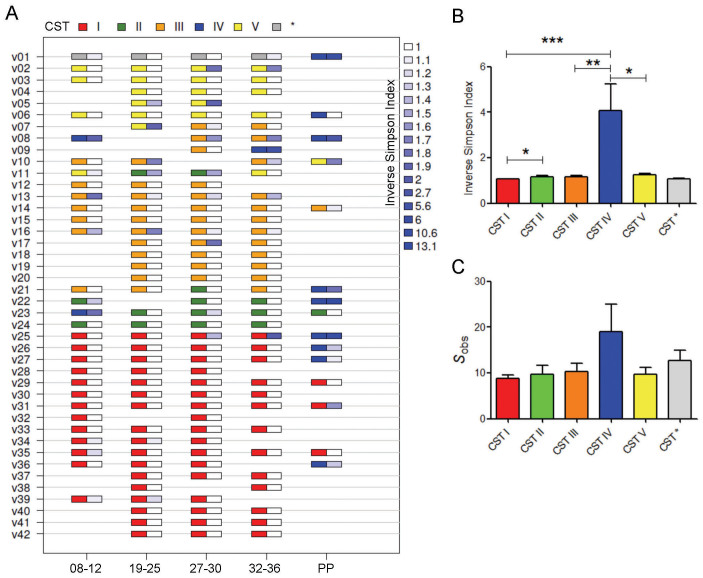
Vaginal community state types (CST) throughout pregnancy and postpartum in a UK population. (A) Longitudinal samples were assigned to CSTs on the basis of ward linkage clustering of microbial species data (CST I, red; CST II, green; CST III, orange, CST IV, blue and CST V, yellow. CST * indicates *Lactobacillus amylovorous* dominated microbiome). Corresponding inverse Simpson indices are presented adjacent (white indicates low diversity, dark blue indicates high diversity). (B) Samples from CST I displayed the lowest diversity as measured by the mean inverse Simpson index whereas CST IV showed significantly higher diversity. No difference in diversity was observed between CST I and CST V. (C) CST IV was associated with increased richness as described by number of species observed. ***P < 0.001, **P < 0.01, *P < 0.05. Kruskall-Wallis test (Dunn's *post hoc*).

The dynamics of vaginal CSTs during pregnancy and postpartum was assessed in longitudinally collected samples (Figure 3A, Table 1, Supplementary Tables 1–3). Pregnant vaginal communities dominated by *L. crispatus* (CST I) had the lowest alpha diversity and richness as well as the greatest stability during pregnancy (Figure 3B and C). In samples where *L. crispatus* was dominant it was the only species seen in 85% of samples, with the other 15% samples showing only very small proportions of other *Lactobacillus* spp. or bacteria from the class Prevotella. Low diversity and richness was also seen in CST V where *L. jensenii* was the dominant species in 70% of samples, with small proportions of other *Lactobacillus* spp. observed in the remaining 30% of samples. Slightly higher alpha diversity and richness was observed in CST II and CST III vaginal microbiomes however, the highest diversity and richness indices were reported for those women with CST IV. While only 2% (3/157) of samples collected throughout pregnancy had a microbiome assigned to CST IV, 60% (9/15) of samples collected postpartum displayed a microbiome consistent with this diverse community state despite having *Lactobacillus* spp. dominated vaginal microbiomes through the duration of pregnancy.

To examine the frequency of each CST between samples collected during pregnancy and those collected 6 weeks post partum, we used a linear mixed model regression modelling (Table 1, Supplementary Tables 1–3). While CST category does not change over the course of pregnancy (Supplementary Table 3) the prevalence of CST-IV is significantly increased postpartum, independent of ethnicity (Table 1). A similar regression using microbial species data showed that the post-partum period is associated with a decrease in *Lactobacillus* spp., particularly *L. crispatus*, and an increase in a number of BV associated species including *Prevotella* spp., *Finegodia magna*, *Streptococcus anginosus* and other rarer species. It should be noted however that in some cases statistical significance was lost post-correction for multiple testing (Supplementary Table 2). Microbial species abundance showed no significant changes over the four time-points assessed during pregnancy (Supplementary Table 1).

### Examination of Ethnic differences in the pregnant and postpartum vaginal microbiome

**Figure 4:**
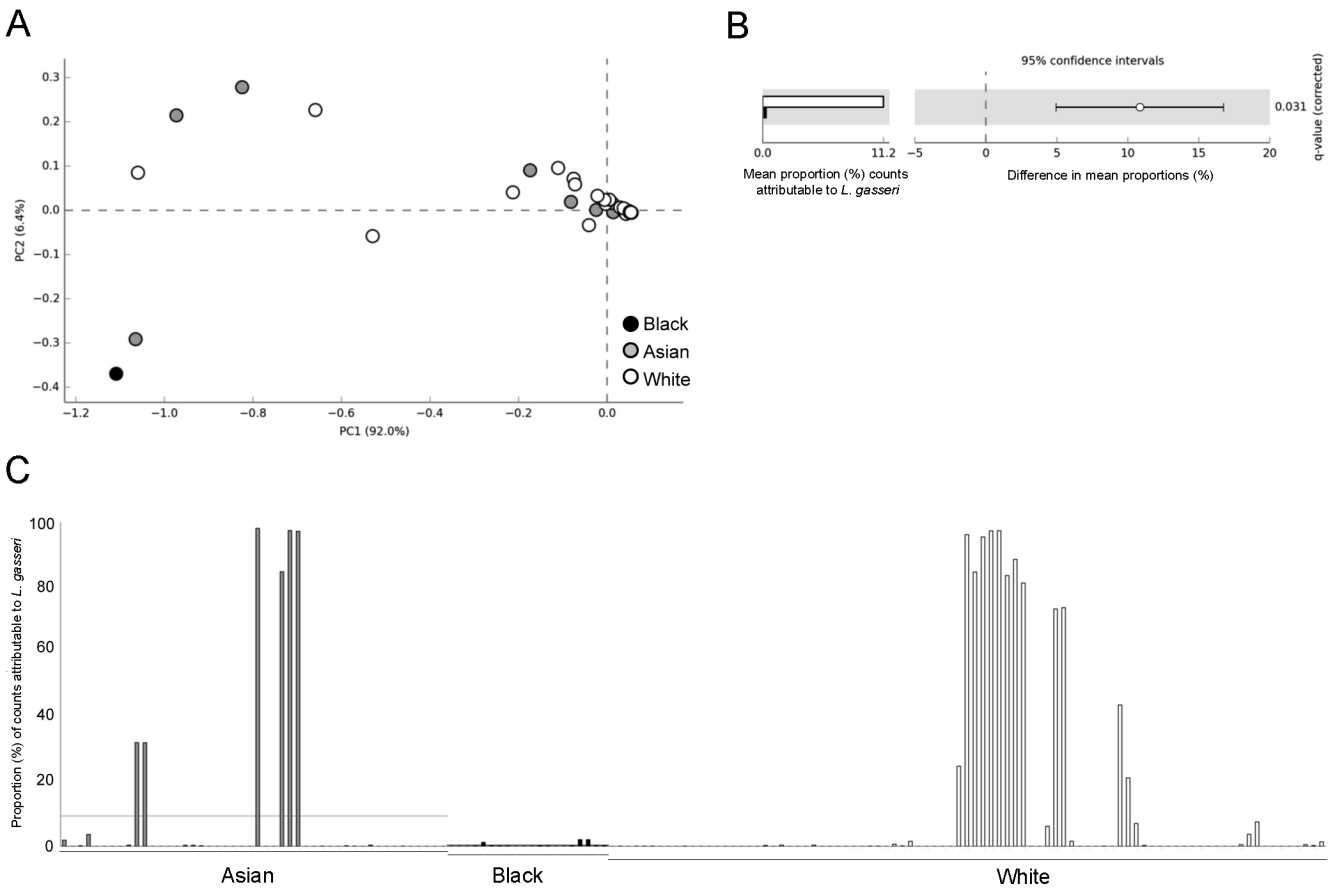
Major microbial community structure changes in the pregnant vagina are independent of ethnicity. (A) Principal component analysis of the vaginal microbiome class data show that high diversity or low diversity is not associated with ethnicity. (B) Levels of *Lactobacillus gasseri* (dominant taxa feature of CST V) are significantly less in black women compared to white women (Welch's t-test with Benjamini-Hochberg FDR correction). (C) Proportion of sequences attributed to *Lactobacillus gasseri* in samples derived from White, Asian and Black ethnicities clearly shows increased frequency of this species in White compared to Black.

To determine if changes in the vaginal microbial community composition in pregnancy and post partum were associated with ethnicity, we analysed the bacterial class structures using PCA. A microbiome consisting of a diverse mixture of bacterial classes typically observed post partum was not specific to any ethnic group (Figure 4A). CSTs I, III and IV were represented by similar proportions of White, Asian and Black ethnicities. However, CST II was not observed in samples collected from Black women and of these, only 1 of 21 (5%) was shown to have a CST V microbiome (Figure 2C and D). Consistent with this, significantly fewer *L. gasseri* (CST II) were detected in samples collected from Black women (<1% mean proportion of sequences) compared to White women (11.2% mean proportion; *q* = 0.031, Welch's t-test with Benjamini-Hochberg FDR correction) (Figure 4B). In total, 6 samples derived from 4 Asian women and 13 samples from 4 White women had a proportion of >20% of total sequences assigned to *L. gasseri*. In contrast, from a total of 20 samples collected from 5 Black women, none contained more than 1% of total sequences attributable to *L. gasseri*. Two bacterial species were detected exclusively in Asian women compared to Black and White women including *L. amylovorus* (detected in 4 samples from the same Asian woman) and *Anaeroglobus germinates*, the latter accounting for less than 1% of the proportion of total sequences within each sample (Supplementary Figure 3).

## Discussion

Our data reveals that the composition of the vaginal microbiome is dynamically restructured in the postpartum period. These findings are consistent with a recently published cross-sectional study of the microbiota of the cervix, posterior fornix and vaginal canal in a limited number (n = 5) of samples collected postpartum^42^. This dynamism provides evidence that oestrogen is likely an important factor in shaping the composition of the vaginal microbiome, particularly during pregnancy^43^,^44^. During pregnancy, placental production of oestrogen causes circulating concentrations to rise dramatically^45^,^46^. Increased levels are thought to increase the proportion of *Lactobacillus* spp. in the vagina through oestrogen-driven maturation of the vaginal epithelium leading to the accumulation of glycogen^47^. Host α-amylase present in vaginal mucosa breaks down glycogen to products including maltose, maltotriose, and maltotetraose that support *Lactobacillus* spp. colonization^48^. Chemical modulation of hormonal levels by oral contraceptives has also been shown to modulate the composition of the vaginal microbiome^49^. During the first week of the postpartum period oestrogen levels fall 100- to 1000-fold^50^,^51^. Any oestrogen-driven *Lactobacillus* spp. dominance of the vaginal microbiome during pregnancy should therefore be dynamically altered during the postpartum period. Our findings support this notion with 40% of the patients assessed 6 weeks postpartum displaying a vaginal microbiome depleted of *Lactobacillus* spp. and enriched with bacterial vaginosis associated species, compared to only 2% during pregnancy. This finding is of particular interest considering the association between dysbiosis of the vaginal microbiome and post-delivery pathologies, particularly postpartum endometritis, which occurs following 1–3% of all deliveries^52^ and is the most common cause of postnatal morbidity 2–10 days post-delivery. The observed shift in the composition of vaginal microbiome postpartum appears to occur irrespective of the community composition observed during pregnancy and is independent of ethnicity. We therefore posit that the rapid reduction of oestrogen levels in the postpartum period leads to a decrease in glycogen and hence, glycogen break down products utilised by lactate-producing bacteria subsequently reducing the community stability and resilience of the vaginal microbiome. This link is consistent with recent data from non-gravid women that shows temporal dynamics in the composition of the vaginal microbiome appear to be hormonally regulated^44^,^53^. However a limitation of these studies and indeed our own, is that direct measurements of oestrogen in the vaginal mucosa were not obtained and thus it is difficult to establish a direct link between hormonal regulation of the vaginal microbiota. It is also likely that alkaline lochial discharge during the post-partum period impedes *Lactobacillus* spp. growth and is thus an important factor in shaping the vaginal microbial composition post-delivery. Discharge rates and volume likely differ between subjects, which may account partly for why 60% of women displayed a microbiome dominated by *Lactobacillus* spp. In keeping with this, Srinivasan and colleagues (2012) have showed that quantities of *L. jensenii* and *L. crispatus* fall during the onset of menstruation^54^. Clearly further studies are required to examine both the causal mechanism and relative contribution of sex hormones such as oestrogen and progesterone in the shaping of the vaginal microbiome during pregnancy and post-delivery.

Analysis of the sequence data using hierarchical cluster analysis showed that the vaginal microbiome of the subjects in this study could be clustered into 5 major groups that are consistent with those CSTs previously described in North American, reproductive-aged women^17^. Although the predominant CST observed during pregnancy in our cohort was CST I (*L. crispatus*), we also identified a number of Asian and White women who had a vaginal microbiome dominated by *L. jensenii* (CST V) or *L. gasseri* (CST II). A vaginal community composition dominated by *L. jensenii* (>80% relative abundance) was observed in only 1 sample collected postpartum from a Black woman. CST II was not observed in samples collected from women of Black ethnicity. In two recent North American studies by Romero and colleagues, the composition of the pregnant vaginal microbiome was reported to be most frequently dominated by *L. iners* (CST III), whereas fewer women presented with *L. crispatus* (CST I) dominated vaginal microbiomes^3^,^55^. A significant contribution of *L. jensenii* (CST V) to the normal pregnancy microbiome was not observed while around 6% of women had a CST II (*L. gasseri*) dominated microbiome. Notably the subject cohorts used in these studies were drawn from a Black ethnic dominated background population. It is possible that sample size limitations and thus random variation effects could account for the apparent increased presence of a *L. jensenii*-dominated microbiome in our cohort. However, in a recent study from Hyman et al, only 1 swab sample from a total of 138 collected from women of multiple ethnic backgrounds were reported as having greater than 1% total sequence reads derived from *L. jensenii*^23^. In contrast, Aagaard and colleagues (2012) reported cross-sectional data showing an enrichment of *L. crispatus*, *L. johnsonii* and *L. jensenii* in the vaginal microbiome with pregnancy, although detailed information regarding ethnicity of the women was not provided^1^. Combined with these recent findings, our results highlight geographical and ethnic differences in the core community structures of the vaginal microbiome during pregnancy. This observation needs to be accounted for when considering the role of the vaginal microbiome in pregnancies with poor outcomes. As previously hypothesised, differences in the vaginal microbial composition between ethnic groups may potentiate their predisposition to bacterial vaginosis and infections during pregnancy due to differences in community resilience, which describes the ability of a given community to resist stresses and perturbations and return to a stable equilibrium^17^,^20^,^56^. For example, a recent report examining the temporal dynamics of the vaginal microbiota and human papillomavirus infection showed that women with a *L. gasseri* dominated microbiome had the fastest HPV remission rate where as those with reduced *Lactobacillus* spp. and increased *Atopobium* had the slowest rate^57^.

Our analysis of the pregnancy and postpartum vaginal microbiome confirms that pregnancy is characterised by an enrichment of *Lactobacillus* spp. However, our data clearly show that specific community state type is not a requisite for an uncomplicated term delivery. Although comparatively rare, we identified some vaginal microbiome samples collected during pregnancy that were *Lactobacillus* spp. deplete and anaerobe-enriched (CST IV) yet still delivered at term. This inconsistency highlights the current poor understanding of how the vaginal microbiome may promote healthy pregnancy outcomes. A detailed assessment of individual host-microbe interactions will be critical for understanding the functional implications of the vaginal microbiome in this context. To date studies have largely aimed to describe vaginal composition during pregnancy. Undoubtedly there exists a complex interplay between the vaginal microbiota and host immune response, both locally and systemically, as well its metabolic milieu. For example, lactic acid enhances the release of IL-1β and IL-8 from vaginal epithelial cells, suggesting a synergistic relationship between inflammatory activation in the host and microbial composition, which is likely dependent on both intrinsic (genetic) and extrinsic (environmental) factors^58^. Moreover, although vaginal epithelial cells produce only L-lactic acid isomer^59^, lactic acid-producing bacteria including *Lactobacillus* spp. produce both the D- and L-lactic acid isomers^60^. Witkin and colleagues (2013) recently reported elevated levels of D-lactic acid in vaginal microbiomes dominated by *L. crispatus* and showed that an increased ratio of D- to L-lactic acid promotes the expression of vaginal extracellular matrix metalloproteinase inducer, which in turn can activate matrix metalloproteinase-8 and may subsequently alter cervical integrity^61^. Such studies designed to improve understanding of the functional relevance of specific vaginal community state types during pregnancy could help clarify recent conflicting reports regarding the association of the vaginal microbiome in preterm birth outcomes. Although one study reported a correlation between a diverse microbiome and preterm birth in cross-sectional data^23^, a longitudinal analysis of the vaginal microbiome in patients destined to deliver preterm failed to show any changes in bacterial taxa associated with spontaneous preterm birth^55^.

In conclusion, our study reveals new insights into biogeographical and ethnic differences that exist between microbial communities in the vaginal microbiome during pregnancy and in the postpartum period. This has implications for future studies designed to explore relationships between the vaginal microbiome and pregnancy outcomes and in particular, highlights the importance of patient-centred individualised treatment strategies designed to modulate the vaginal microbiome to promote health during pregnancy and beyond.

## Author Contributions

Designed project: D.A.M., M.C., J.K.N., J.R.M. and P.R.B. Collected samples: M.C., L.K., S.A., R.B. and T.G.T. Performed experiments: D.A.M, M.C. and Y.L. Analysed data: D.A.M., A.S., N.A., B.C.L., E.H., J.K.N and J.R.M. Generated figures and tables: D.A.M., A.S., N.A., B.C.L. and J.R.M. Wrote manuscript: D.A.M. and P.R.B. All authors critically reviewed the manuscript.

## Supplementary Material

Supplementary Information Supplementary Information

## Figures and Tables

**Table 1:** Linear mixed model regression analysis for exploring the association between CSTs and pregnancy status. To assess if CSTs change significantly postpartum, a binary time variable was created with time = 0 for measurements during pregnancy and time = 1 for postpartum measurements and linear regression performed for each CST. Time was regressed against CST adjusted for ethnicity and patient ID, whereby patient ID is modelled as a random effect to account for the correlation between samples of the same individual. Coefficient estimates and standard deviation are presented alongside t-values, *P*-values and *q*-values for the model. The results indicate that the prevalence of CST-IV is significantly increased postpartum

CST	Pregnancy	Postpartum	Estimate	Std. Error	t value	*P* value	*Q* value
**I**	60 (43%)	3 (20%)	0.093	0.049	1.894	5.8E-02	1.7E-01
**II**	13 (9%)	1 (7%)	0.022	0.085	0.267	7.9E-01	7.9E-01
**III**	42 (30%)	1 (7%)	0.111	0.054	2.051	4.0E-02	1.6E-01
**IV**	3 (2%)	9 (60%)	−0.718	0.069	−10.271	9.5E-25	4.7E-24
**V**	20 (14%)	1 (7%)	0.048	0.072	0.671	5.0E-01	7.9E-01

## Notes

**Accession codes:** Public access to sequence data and accompanying metadata can be obtained at the European Nucleotide Archive’s (ENA) Sequence Read Archive (SRA) (accession number PRJEB7703).
