# Supplementary material for: The vaginal microbiome during pregnancy and the postpartum period in a European population
Source: Sci Rep. 2015 Mar 11;5:8988. doi: 10.1038/srep08988 (PMC4355684; doi:10.1038/srep08988)
Supplement: Supplementary Information [file srep08988-s1.pdf]

The vaginal microbiome during pregnancy and the postpartum period in a European population.

David A. MacIntyre[1*], Manju Chandiramani[1], Yun S Lee[1], Lindsay Kindinger[1], Ann Smith[2], Nicos Angelopoulos[3], Benjamin C Lehne[4], Shankari Arulkumaran[1], Richard Brown[1], Tiong Ghee Teoh[5], Elaine Holmes[6], Jeremy K Nicoholson[6,7], Julian R Marchesi[3,6] and Phillip R Bennett[1].

SUPPLEMENTARY INFORMATION

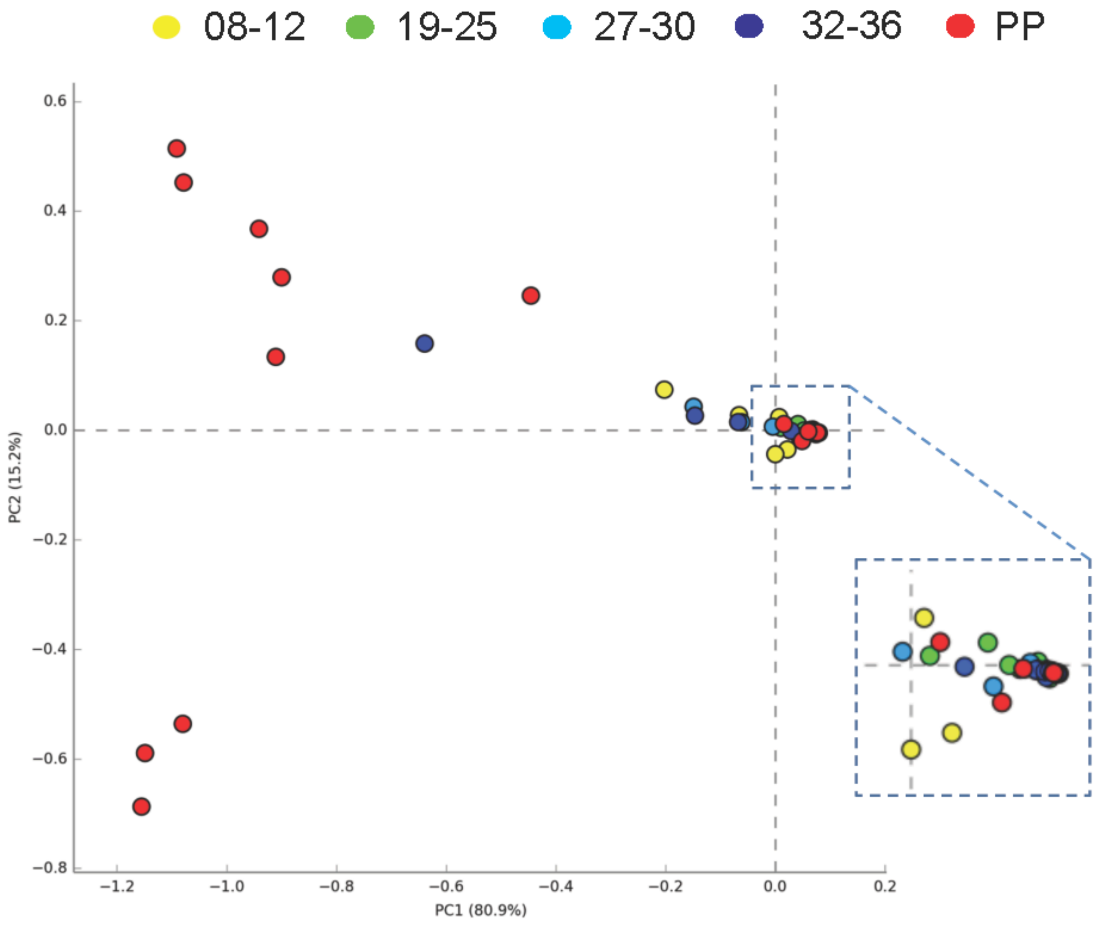

Supplementary Figure 1. PCA of bacterial genera data collected from patients at 8-12 (yellow), 19-25 (green), 27-30 (light blue) and 32-36 (dark blue) weeks gestation or 6 weeks post partum (PP; red).

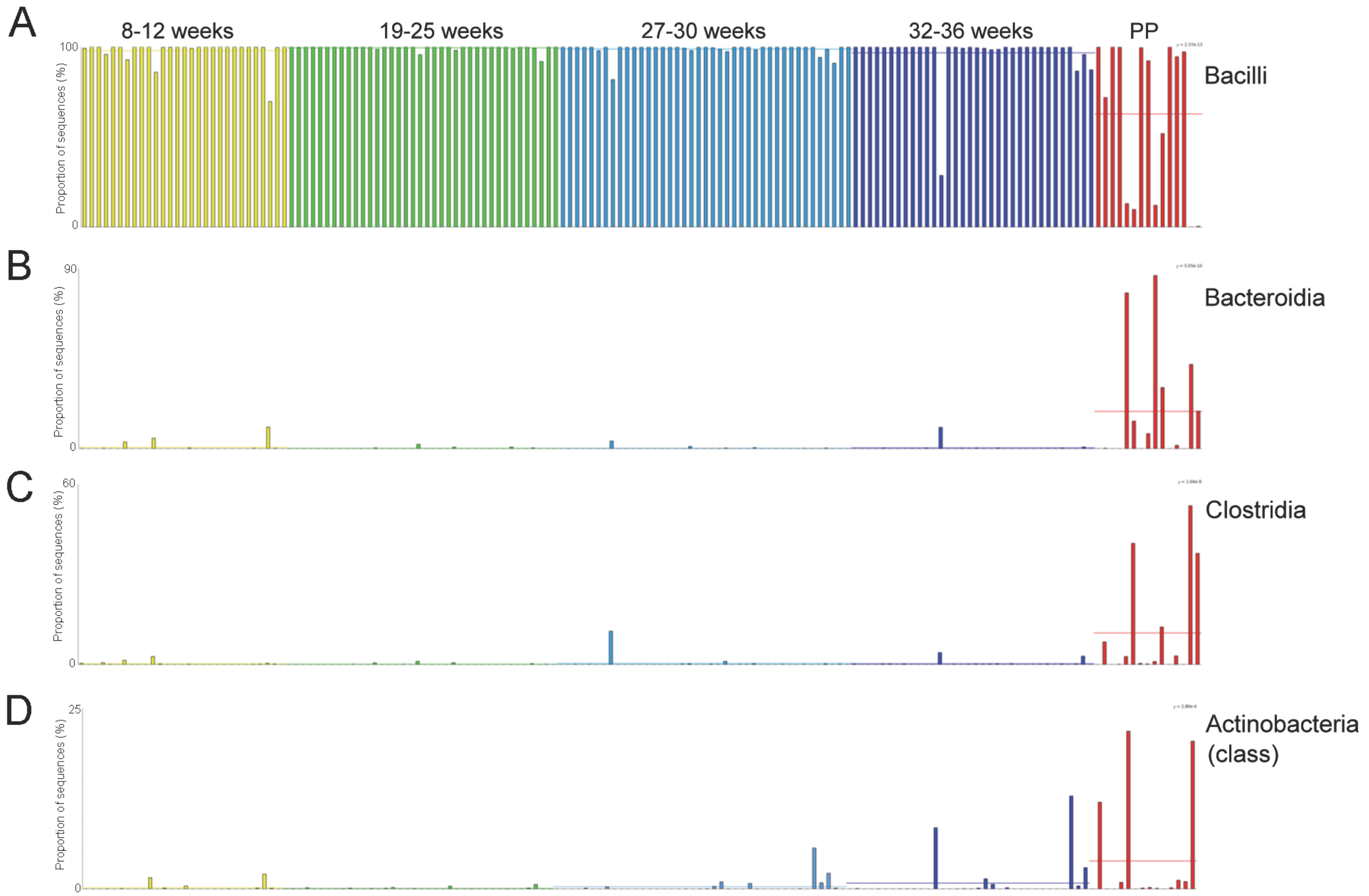

Supplementary Figure 2. Relative abundance of bacterial classes in cross sectional data collected from patients at 8-12 (yellow), 19-25 (green), 27-30 (light blue) and 32-36 (dark blue) weeks gestation or 6 weeks post partum (PP; red). Data is presented to as proportion of sequences assigned to bacterial classes **(A)** Bacilli, **(B)** Bacteroidia, **(C)** Clostridia or **(D)** Actinobacteria.

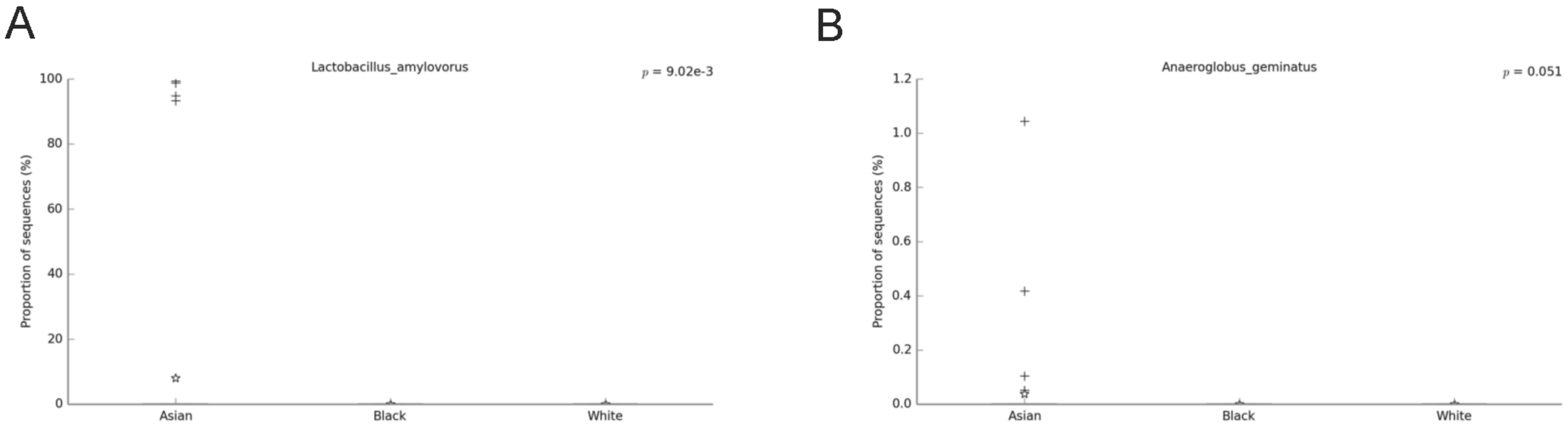

Supplementary Figure 3. Bacterial species detected predominately in Asian samples. Box plot describing proportion of sequences derived from **(A)** *Lactobacillus amylovorus* and **(B)** *Anaeroglobus germinatus.* Star indicates mean proportion of group.

Supplementary Table 1. Linear mixed model regression analysis for examining bacterial species abundance across pregnancy time points. To test if bacterial species abundance changes significantly over the course of pregnancy we excluded postpartum measurements and performed a linear regression for each microorganism. Abundances were log-transformed and regressed against time and adjusted for ethnicity and patient ID, whereby patient ID is modelled as a random effect to account for the correlation between samples of the same individual. “NA” indicates a lack of convergence in the model because of too few data-points. Fold changes are reported as NAs if values in one of the two groups are 0 or if the corresponding regression-metrics are non-zero.

Species	Fold Change	Estimate	Std. Error	t value	P value	Q value
Lactobacillus_crispatus	1.2802	0.0056	0.0260	0.2168	8.3E-01	1.0E+00
Lactobacillus_iners	-1.3837	-0.0305	0.0332	-0.9179	3.6E-01	1.0E+00
Lactobacillus_jensenii	-1.0210	-0.0290	0.0397	-0.7314	4.6E-01	1.0E+00
Lactobacillus_gasseri	-1.1092	0.0556	0.0481	1.1555	2.5E-01	1.0E+00
Lactobacillus_amylovorus	1.1531	0.0031	0.0030	1.0304	3.0E-01	1.0E+00
Prevotella_timonensis	-3.8382	0.0086	0.0334	0.2578	8.0E-01	1.0E+00
Prevotella_bivia	4.5640	-0.0134	0.0292	-0.4591	6.5E-01	1.0E+00
Streptococcus_agalactiae	38.8851	-0.0269	0.0195	-1.3787	1.7E-01	1.0E+00
Unclassified	-1.8171	-0.0023	0.0232	-0.1007	9.2E-01	1.0E+00
Atopobium_vaginae	-12.2966	0.0411	0.0314	1.3085	1.9E-01	1.0E+00
Finegoldia_magna	-1.0441	0.0143	0.0251	0.5692	5.7E-01	1.0E+00
Lactobacillus_delbrueckii	42.8582	-0.0147	0.0126	-1.1611	2.5E-01	1.0E+00
Sneathia_sanguinegens	NA	0.0616	0.0628	0.9809	3.3E-01	1.0E+00
Prevotella_disiens	2.3448	-0.0059	0.0083	-0.7096	4.8E-01	1.0E+00
Prevotella_amnii	NA	NA	NA	NA	NA	NA
Streptococcus_anginosus	1.7241	0.0087	0.0184	0.4716	6.4E-01	1.0E+00
Ureaplasma_parvum	5.3038	-0.0083	0.0178	-0.4690	6.4E-01	1.0E+00
Anaerococcus_vaginalis	-7.6765	0.0221	0.0153	1.4481	1.5E-01	1.0E+00
Aerococcus_christensenii	-3.7557	0.0331	0.0308	1.0724	2.8E-01	1.0E+00
Prevotella_buccalis	NA	-0.0278	0.0200	-1.3915	1.6E-01	1.0E+00
Dialister_micraerophilus	-1.5993	0.0061	0.0121	0.5040	6.1E-01	1.0E+00

Prevotella_corporis	NA	-0.0120	0.0117	-1.0274	3.0E-01	1.0E+00
Dialister_propionicifaciens	4.6897	-0.0092	0.0153	-0.6026	5.5E-01	1.0E+00
Veillonella_montpellierensis	1.1724	0.0035	0.0105	0.3342	7.4E-01	1.0E+00
Peptoniphilus_lacrimalis	NA	0.0056	0.0043	1.3124	1.9E-01	1.0E+00
Lactobacillus_salivarius	NA	NA	NA	NA	NA	NA
Staphylococcus_devriesei	-1.7059	-0.0018	0.0094	-0.1878	8.5E-01	1.0E+00
Anaerococcus_lactolyticus	NA	0.0085	0.0113	0.7513	4.5E-01	1.0E+00
Staphylococcus_capitis	1.1724	-0.0020	0.0064	-0.3216	7.5E-01	1.0E+00
Peptoniphilus_harei	NA	-0.0087	0.0108	-0.8073	4.2E-01	1.0E+00
Peptostreptococcus_anaerobius	NA	NA	NA	NA	NA	NA
Peptoniphilus_gorbachii	NA	NA	NA	NA	NA	NA
Fusobacterium_nucleatum	NA	-0.0070	0.0079	-0.8837	3.8E-01	1.0E+00
Gardnerella_vaginalis	1.3856	0.0000	0.0099	-0.0036	1.0E+00	1.0E+00
Campylobacter_ureolyticus	NA	0.0059	0.0080	0.7415	4.6E-01	1.0E+00
Peptococcus_niger	NA	NA	NA	NA	NA	NA
Corynebacterium_pyruviciproducens	-5.1176	0.0026	0.0093	0.2781	7.8E-01	1.0E+00
Streptococcus_gallolyticus	NA	NA	NA	NA	NA	NA
Porphyromonas_bennonis	NA	0.0008	0.0020	0.4159	6.8E-01	1.0E+00
Prevotella_denticola	NA	NA	NA	NA	NA	NA
Anaeroglobus_geminatus	NA	-0.0122	0.0081	-1.5024	1.3E-01	1.0E+00
Anaerococcus_murdochii	NA	-0.0076	0.0082	-0.9244	3.6E-01	1.0E+00
Propionibacterium_acnes	NA	-0.0004	0.0028	-0.1328	8.9E-01	1.0E+00
Actinomyces_europaeus	NA	NA	NA	NA	NA	NA
Scardovia_wiggsiae	NA	NA	NA	NA	NA	NA
Murdochiella_asaccharolytica	NA	0.0002	0.0037	0.0600	9.5E-01	1.0E+00
Rare OTUs	-1.7059	0.0195	0.0175	1.1147	2.6E-01	1.0E+00

Supplementary Table 2. Linear mixed model regression analysis for exploring the association between microbial species abundance and pregnancy status. To examine if microorganism abundance changes significantly after pregnancy, a binary time variable was created with time=0 for measurements during pregnancy and time=1 for postpartum measurements. Using this binary time variable we performed a linear regression for each microorganism. Abundances were log-transformed and regressed against time adjusted for ethnicity and patient ID, whereby patient ID is modelled as a random effect to account for the correlation between samples of the same individual. Fold changes are reported as NAs if values in one of the two groups are 0 or if the corresponding regression-metrics are non-zero.

Species	Fold Change	Estimate	Std. Error	t value	*P* value	*Q* value
Lactobacillus_crispatus	-2.2915	-0.6555	0.2883	-2.2739	2.3E-02	6.7E-01
Lactobacillus_iners	-2.4702	-0.2210	0.1709	-1.2936	2.0E-01	8.9E-01
Lactobacillus_jensenii	-2.2858	-0.4816	0.2539	-1.8971	5.8E-02	8.9E-01
Lactobacillus_gasseri	-1.4661	-0.1517	0.2472	-0.6135	5.4E-01	8.9E-01
Lactobacillus_amylovorus	-260.6690	-0.0418	0.0801	-0.5225	6.0E-01	8.9E-01
Prevotella_timonensis	45.2014	0.9192	0.3169	2.9003	3.7E-03	1.3E-01
Prevotella_bivia	178.2422	0.5947	0.3345	1.7781	7.5E-02	8.9E-01
Streptococcus_agalactiae	141.4718	0.4491	0.2961	1.5167	1.3E-01	8.9E-01
Unclassified	11.4152	1.0624	0.2972	3.5745	3.5E-04	1.4E-02
Atopobium_vaginae	-38.1866	-0.0863	0.0917	-0.9408	3.5E-01	8.9E-01
Finegoldia_magna	21.9414	0.5846	0.1885	3.1007	1.9E-03	6.9E-02
Lactobacillus_delbrueckii	-167.4296	-0.0484	0.0638	-0.7578	4.5E-01	8.9E-01
Sneathia_sanguinegens	NA	-0.0278	0.0526	-0.5286	6.0E-01	8.9E-01
Prevotella_disiens	153.6941	0.4531	0.2028	2.2344	2.5E-02	7.1E-01
Prevotella_amnii	NA	NA	NA	NA	NA	NA
Streptococcus_anginosus	302.9333	0.7621	0.2808	2.7141	6.6E-03	2.1E-01
Ureaplasma_parvum	2.6371	0.0753	0.1074	0.7008	4.8E-01	8.9E-01
Anaerococcus_vaginalis	31.6893	0.6372	0.2194	2.9039	3.7E-03	1.3E-01
Aerococcus_christensenii	-2.7097	-0.0150	0.1089	-0.1375	8.9E-01	8.9E-01
Prevotella_buccalis	3.7013	0.3226	0.1237	2.6074	9.1E-03	2.8E-01
Dialister_micraerophilus	55.0788	0.4407	0.1616	2.7278	6.4E-03	2.1E-01

Prevotella_corporis	59.4701	0.2892	0.2042	1.4166	1.6E-01	8.9E-01
Dialister_propionicifaciens	51.6722	0.3942	0.1713	2.3010	2.1E-02	6.4E-01
Veillonella_montpellierensis	NA	-0.0113	0.0330	-0.3419	7.3E-01	8.9E-01
Peptoniphilus_lacrimalis	355.0000	0.3717	0.1782	2.0860	3.7E-02	8.9E-01
Lactobacillus_salivarius	NA	0.0201	0.0061	3.3051	9.5E-04	3.6E-02
Staphylococcus_devriesei	15.7778	0.1133	0.1071	1.0584	2.9E-01	8.9E-01
Anaerococcus_lactolyticus	36.8148	0.3669	0.1932	1.8987	5.8E-02	8.9E-01
Staphylococcus_capitis	11.5704	0.1093	0.0709	1.5415	1.2E-01	8.9E-01
Peptoniphilus_harei	46.5444	0.2067	0.1333	1.5504	1.2E-01	8.9E-01
Peptostreptococcus_anaerobius	NA	0.3284	0.0473	6.9379	4.0E-12	1.8E-10
Peptoniphilus_gorbachii	NA	0.1000	0.0253	3.9524	7.7E-05	3.2E-03
Fusobacterium_nucleatum	27.4533	0.0903	0.1253	0.7208	4.7E-01	8.9E-01
Gardnerella_vaginalis	25.7885	0.1254	0.0937	1.3385	1.8E-01	8.9E-01
Campylobacter_ureolyticus	14.8762	0.1085	0.0908	1.1953	2.3E-01	8.9E-01
Peptococcus_niger	NA	0.1531	0.0287	5.3412	9.2E-08	4.0E-06
Corynebacterium_pyruviciproducens	1.9930	0.0794	0.0406	1.9561	5.0E-02	8.9E-01
Streptococcus_gallolyticus	NA	NA	NA	NA	NA	NA
Porphyromonas_bennonis	757.3333	0.2864	0.1429	2.0044	4.5E-02	8.9E-01
Prevotella_denticola	NA	NA	NA	NA	NA	NA
Anaeroglobus_geminatus	-1.1268	0.0378	0.0353	1.0687	2.9E-01	8.9E-01
Anaerococcus_murdochii	24.6133	0.1225	0.0773	1.5846	1.1E-01	8.9E-01
Propionibacterium_acnes	137.2667	0.1311	0.1008	1.2999	1.9E-01	8.9E-01
Actinomyces_europaeus	NA	0.0795	0.0242	3.2885	1.0E-03	3.7E-02
Scardovia_wiggsiae	NA	0.1006	0.0298	3.3781	7.3E-04	2.8E-02
Murdochiella_asaccharolytica	50.4889	0.1511	0.0832	1.8150	7.0E-02	8.9E-01
Rare OTUs	6.9502	0.4082	0.1079	3.7842	1.5E-04	6.3E-03

Supplementary Table 3. Linear mixed model regression analysis for examining CSTs across pregnancy timepoints. To test if CSTs change significantly over the course of pregnancy we excluded postpartum measurements and performed a linear regression for each CST. A CST indicator variable was created where CST=1 for samples that could be assigned to the given CST and CST=0 for all other samples. Time was regressed against CST adjusted for ethnicity and patient ID, whereby patient ID is modelled as a random effect to account for the correlation between samples of the same individual.

CST	08-12	19-25	27-30	32-36	Estimate	Std. Error	t value	*P* value	*Q* value
**1**	13 (46%)	17 (46%)	17 (43%)	13 (37%)	0.1553	0.1868	0.8312	4.1E-01	9.3E-01
**2**	2 (7%)	3 (8%)	5 (12%)	3 (9%)	-0.1253	0.3163	-0.3963	6.9E-01	9.3E-01
**3**	7 (25%)	11 (30%)	12 (30%)	12 (36%)	-0.2094	0.2031	-1.0314	3.0E-01	9.3E-01
**4**	2 (7%)	0 (0%)	0 (0%)	1 (3%)	0.6234	0.6319	0.9865	3.2E-01	9.3E-01
**5**	4 (14%)	6 (16%)	5 (12%)	5 (15%)	0.0224	0.2668	0.0841	9.3E-01	9.3E-01
